# Macrosynteny analysis between *Lentinula edodes* and *Lentinula novae-zelandiae* reveals signals of domestication in *Lentinula edodes*

**DOI:** 10.1038/s41598-021-89146-y

**Published:** 2021-05-10

**Authors:** Christopher Alan Smith

**Affiliations:** Manaaki Whenua – Landcare Research, Auckland, New Zealand

**Keywords:** Eukaryote, Evolutionary biology, Genome, Genomics, Fungal evolution, Fungal genomics

## Abstract

The basidiomycete fungus *Lentinula novae-zelandiae* is endemic to New Zealand and is a sister taxon to *Lentinula edodes*, the second most cultivated mushroom in the world. To explore the biology of this organism, a high-quality chromosome level reference genome of *L. novae-zelandiae* was produced. Macrosyntenic comparisons between the genome assembly of *L. novae-zelandiae*, *L. edodes* and a set of three genome assemblies of diverse species from the Agaricomycota reveal a high degree of macrosyntenic restructuring within *L. edodes* consistent with signal of domestication. These results show *L. edodes* has undergone significant genomic change during the course of its evolutionary history, likely a result of its cultivation and domestication over the last 1000 years.

## Introduction

The genus *Lentinula* from the Basidiomycete order Agaricales, has a global distribution and consists of 7 described species, one of which is the well-known gourmet edible mushroom *Lentinula edodes*, colloquially known as Shiitake. New Zealand is home to a single endemic species of *Lentinula* that represents the monophyletic lineage *Lentinula novae-zelandiae*. Research investigating the biogeography and phylogenetic history of the *Lentinula* genus strongly supports a Laurasian descent for *L. novae-zelandiae*, with a single long-distance dispersal event from Australia sometime within the last 8 million years^1^. This data confirms earlier research that shows *L. novae-zelandiae* belongs to a monophyletic clade found only in New Zealand. Aside from these articles, little research has been undertaken on L*. novae-zelandiae*.

In contrast to this, *L. edodes* has a large natural range spanning mainland China and an even larger expanded range as it is now the second most cultivated mushroom in the world^2^. Known as the Shiitake mushroom after its common host, the Shii Tree (*Castanopsis cuspidate)*, this fungus has been cultivated in China for at least 900–1000 years, with wild foraging being undertake for at least 1800 years^2^. This process has resulted in a number of highly productive domesticated strains that are used to produce what has become the second largest mushroom food crop in the world^3–6^.

The genomic impact of domestication has been comparatively well studied within the animal and plant kingdoms compared with the fungal kingdom^7^, although some research has been undertaken^4,7–12^. The outcome of this work has identified several features associated with domestication, such as interspecific hybridization events, horizonal gene transfer events, copy number variations, genome decay, and chromosomal rearrangements. Furthermore, a large body of evidence exists showing that mobile genetic elements can play a crucial role in shaping the genomic architecture of an organism regardless of whether it has undergone a domestication process^11,13^. No work to date has investigated whether there is any genomic signal of domestication in the *Lentinula* genus.

Species within the genus *Lentinula*, and in fact most Basidiomycete fungi, exist in the dikaryotic state during vegetative growth^14^. This is a life stage where each cell is home to two nuclei, one from each of the parental strains. When the fungus is preparing to produce fruit bodies, the two separate nuclei fuse in a process known as karyogamy, followed by meiosis. This process results in four daughter spores produced through sexual recombination. In brief, during meiosis homologous chromosomes pair, replicate, then separate. The pairing of homologous chromosomes allows for crossing-over events in which genetic material is exchanged between them^14^. This crossing-over is one of the sources of genetic variation derived through sexual recombination. It can even result in entire arms of a chromosome pair being swapped between homologous chromosomes in extreme cases.

Non-homologous cross-over events, known as ectopic recombination, are likely to occur between repetitive regions within homologous chromosomes which can result in chromosome length polymorphisms^15–17^. Ectopic recombination between non-homologous chromosomes however is rare^15–17^. Research conducted in the 1990′s showed that chromosome length polymorphisms (CLP) existed within species from a number of different fungal lineages and suggested that this was a widespread phenomenon^15–17^. The results from this work show that crosses between strains with CLP’s show progeny with CLP’s intermediate of the parental strains and that crosses between non-polymorphic strains did not produce progeny with CLP’s. Due to the technical methodology at the time the authors were unable to verify whether the CLP’s observed were due to ectopic recombination between repetitive elements on non-homologous chromosomes or homologous pairing between polymorphic chromosomes. The authors do suggest it is highly likely the latter and not the former due to the progeny results between strains with CLP and non-polymorphic strains^16^. No research in this space has been undertaken within the Omphalotaceae or the genus *Lentinula*.

The importance of Shiitake as a food crop has led to efforts to produce a range of high-quality genomic resources, with the current reference genome assembly on NCBI’s GenBank created from Pacific Biosciences long-read technology^18^ and a further six other genome assemblies for *L. edodes* available for public research. In parallel to this, at least seven efforts have been made to produce genetic linkage maps of *L. edodes* from 2002 through to 2019^19–25^, which has resulted in the identification of 11–14 linkage groups. Work by many of the same authors has also developed quantitative trait loci (QTL) associated with important agronomic traits^3,6,21,26^. Furthermore, RNA-Seq experiments have been conducted to understand how Shiitake responds to different environments and treatments^27–29^.

With the advent of third generation long-read sequencing technologies there has been an explosion of new bioinformatic tools to process these data as well as a wealth of new research using this technology. This has allowed for the production of genome assemblies with high levels of contiguity, something previously difficult and expensive to attain with second generation short-read sequencing technologies. These highly contiguous genomes allow for exploration of a number of areas that were previously intractable, such as: large-scale genome structure analysis, known as macrosynteny^30^; and longitudinal studies investigating how genome structure changes over time^31^. Large-scale genome synteny analysis is difficult to undertake with fragmented genomes, with previous efforts focused on specific loci and the ordering of the genes; this is known as microsynteny^32–35^.

Macrosynteny analysis of fungal genomes is limited. While some work^30,36^ has been done in this area in the Agaricales, there is much still to be done. Exploration of these macrosyntenic relationships findings so far highlight a large degree of conserved macrosyntenic structure within the order Agaricales, with an early study finding extensive synteny between the model organism *Coprinopsis cinerea* and the mycorrhizal agaric *Laccaria bicolor*^36^. A recent article found a similar degree of conserved macrosynteny between the edible mushrooms *Agrocybe cylindracea* and *Agrocybe aegerita* and the toxic mushroom *Galerina marginata*^30^.

The monophyletic nature of the *Lentinula novae-zelandiae* lineage combined with the geographic isolation of this species make it a prime candidate for comparative genomic analysis with *L. edodes*. A high-quality chromosome scale genome assembly of *L. novae-zelandiae* was created to be used as the lynchpin for macrosyntenic analysis between it and *L. edodes* to explore whether the domestication of *L. edodes* has resulted in any changes at the chromosome level.

## Results

### Genome assembly and annotation of* Lentinula novae-zelandiae* ICMP 18003

The genome of *L. novae-zelandiae* ICMP 18003 was assembled using 8 Gb of base called long-read MinION data and 18 Gb of paired-end short-read Illumina data, to an average depth of 127X and 137X coverage respectively following quality control. Analysis of the illumina dataset with GenomeScope reported a haploid genome length between 38.58 to 38.63 Mb, consisting of 5.88 to 5.89 Mb of repetitive content and with a heterozygosity of 0.871% to 0.879%. The model fit for these metrics was 95.25% to 96.98%.

The assembly pipeline produced a chromosome scale genome assembly consisting of 17 scaffolds with a total genome size of 48.9 Mb. The assembled genome had a GC content of 46.49%, an N50 of 4,832,147 bp and a L50 of 4, with the largest scaffold being 8,122,969 nucleotides long. Of these 17 scaffolds, the second-longest scaffold is a fully assembled chromosome capped with telomeric sequences on each end and with only a single gap in the scaffold. A further 13 sequences had at least one end capped by telomeric sequence. These telomeric sequences consisted of the repeating motif of TTAGGGG, with between 26 and 31 repetitions, with nucleotide lengths ranging from 183 to 220 bp.

Analysis with BUSCO reported the *L. novae-zelandiae* ICMP 18003 genome assembly has a completeness score of 96.5%. This consists of 3,636 complete single-copy BUSCOs out of a total of 3870, with 98 duplicated BUSCOs and 109 missing BUSCOs.

### Genome assembly annotation

The *L. edodes* B17 and *L. novae-zelandiae* ICMP 18003 genome assemblies had their repetitive content analysed as part of the assembly pipeline. In total the *L. edodes* B17 genome had 24.9% identified as repetitive elements and the *L. novae-zelandiae* ICMP 18003 had 31.82% of its genome identified as repetitive elements. The larger repetitive content of the *L. novae-zelandiae* ICMP 18003 genome compared to the *L. edodes* B17 genome was primarily due to an increase in the number of LTR elements, with significant increases in the number of Gypsy DIRS1 elements accounting for the large bulk of the LTR elements, with the remainder mostly made up of Type 1 Copia elements. There are more than 1000 unclassified repetitive elements identified within the *L. novae-zelandiae* ICMP 18003 assembly than compared with the *L. edodes* B17 genome assembly.

The Funannotate pipeline produced a set of high-quality gene models for both the *L. novae-zelandiae* ICMP 18003 genome and the *L. edodes* B17 genome, with 12,443 gene models predicted for *L. novae-zelandiae* ICMP 18003 and 11,999 for *L. edodes* B17.

### Comparative genomics and macrosynteny

#### Agaricomycete reference genomes

One *L. edodes* genome was identified as suitable for macrosyntenic analysis; the representative genome assembly of *L. edodes*, assembly GCA_001562095.1 identified as strain B17^18^. This genome was selected as the long-read based genome assembly as it was assembled with FALCON^37^ using 61X coverage of long-read data produced via a PacBio RS II system. This assembly was further scaffolded using 120X coverage of long-mated pair reads (5-kb and 10-kb libraries) as well as 86.1X coverage of short-insert reads (500-bp library) with the software SSPACE and GapCloser^18^.

NCBI’s GenBank database yielded 270 genome assemblies for within the Agaricomycetes. Of those, six genome assemblies were identified as being chromosome scale genome assemblies; *Agaricus bisporus* ASM30057v2, *Flammulina velutipes* Fv1.0, *Hericium erinaceus* HeCS-4_2.0, *Pleurotus ostreatus* 03989_v2, *Pyrrhoderma noxium* ASM228747v2 and *Trametes hirsuta* TraHir072. In addition to this, the *C. cinerea* CC3 assembly was selected as a candidate as it is considered a chromosome-scale genome assembly and syntenic analysis has been undertaken on it previously^36^. Furthermore, work done in a recent article^38^ on the genus *Armillaria* produced 11 putative chromosomes for *A. ostoyae*, bringing the total number of chromosome scale assemblies within the Agaricomycetes to eight. The genome assemblies of *C. cinerea* CC3 and *P. noxium* had predicted gene sets and as such were deemed suitable for macrosyntenic analysis.

The *L. edodes* B17 genome had five telomeric regions identified. Of these, three are located embedded within the scaffold in which they were identified, with only two capping the end of a scaffold. None of the embedded telomeric sequences had flanking assembly gaps. The telomeric sequences in the B17 genome ranged from 10 to 25 repeats of the telomeric motif and had nucleotide lengths from 73 to 179 bp. No telomeric regions were identified within the *C. cinerea* CC3 genome. The *P. noxium* genome assembly had 13 telomeric regions identified, all at terminal ends of assembled scaffolds. A summary of genome assembly metrics identified telomeric regions can be found in Supplementary Table 1.

#### Macrosynteny analysis

Pairwise macrosynteny analysis was conducted using both SynChro and Satsuma2 on the following genomes: *C. cinerea* CC3*, L. edodes* B17, *L. novae-zelandiae* ICMP 18003 and *P. noxium*.

A summary of the quantitative results from analysis with SynChro can be found in Supplementary Table 2. In general, SynChro found 67.23% average similarity between syntenic homologs across assessed genome assemblies, except for the *Lentinula* species, which shared 86.21% average similarity. The average number of genes per syntenic block ranged from 4.73 through to 28.91; however, the *Lentinula* species skew that result with an average of 28.91 genes per block, whereas on average all the other pairwise comparisons had an average of 5.56 genes per block. The number of syntenic blocks identified between pairwise comparisons ranged from 361 to 715, with an average of 546.83 syntenic blocks per pairwise comparison. Interestingly, the fewest syntenic blocks were found between *L. edodes* B17 and *L. novae-zelandiae* ICMP 18003 at 361 blocks, however this appears to be due to the number of genes per syntenic block for those comparisons.

Supplementary Table 2 shows how many times two consecutive blocks from one genome were found on the same chromosome of the compared genome. These data provide insights into the macrosyntenic relationships between the pairwise species comparisons. Comparisons between the *L. edodes* B17 genome assembly and any of the other analysed genomes showed a stark difference compared with the pairwise comparisons between the rest of the compared species.

For example, *L. edodes* B17 when compared against *C. cinerea* showed 416 sets of two consecutive blocks of the *L. edodes* genome were found within the *C. cinerea* genome; yet the *P. noxium* genome when compared against *C. cinerea* in the same manner showed 668 blocks, despite it being from a basal order to that which *L. edodes* is found. When comparing *L. novae-zelandiae* to the *L. edodes* B17 genome, only 166 syntenic blocks are identified. In contrast, *L. novae-zelandiae* shows 573 consecutive blocks of its genome are found on the same chromosomes within the *C. cinerea* genome and 402 consecutive blocks shared between it and *P. noxium*.

The ordering of the syntenic blocks identified across pairwise comparisons also matches this trend. Visualization of the macrosyntenic relationships between the pairwise comparisons of genome assemblies with Circos highlight the above-stated trend. These plots readily show the high level of conservation of macrosyntenic structure in all comparisons except for those with *L. edodes*. In these plots the colored ribbon connections between scaffolds represent syntenic blocks, with the width of the connection points scaled to the size of the syntenic block. For each plot the ribbons have been labelled according to one of the genomes as stated in the legend for each. This allows for identifying rearrangements between the query and target genome.

**Figure 1:**
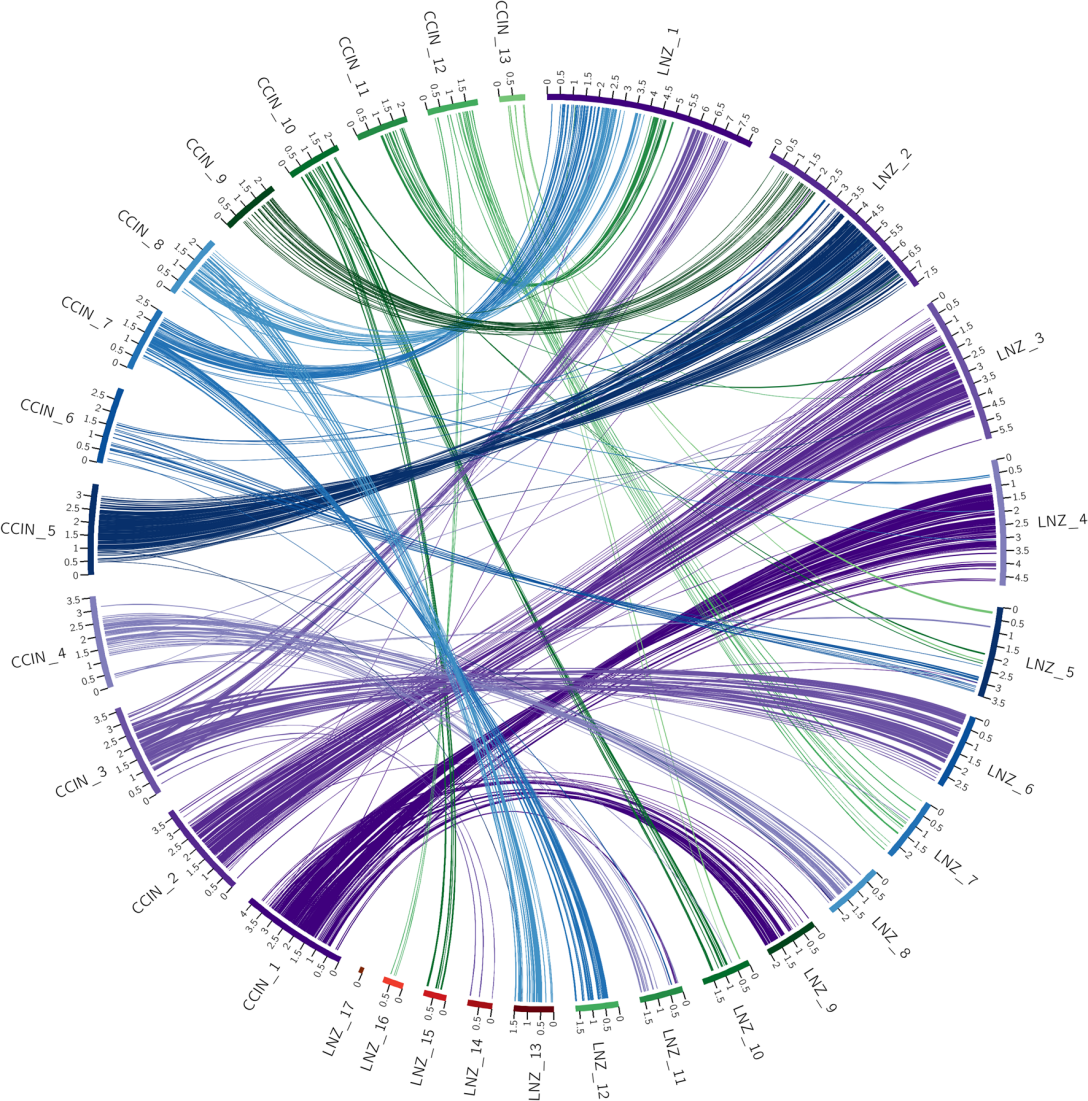
Circos plot of shared syntenic regions between *C. cinerea* and *L. novae-zelandiae* ICMP 18003. Syntenic regions are connected with colored ribbons scaled to the size of the syntenic regions. These ribbons match the colors of the *C. cinerea* scaffold blocks. Genomic scaffold lengths are in Mb and are indicated by the numerals on each scaffold.

**Figure 2:**
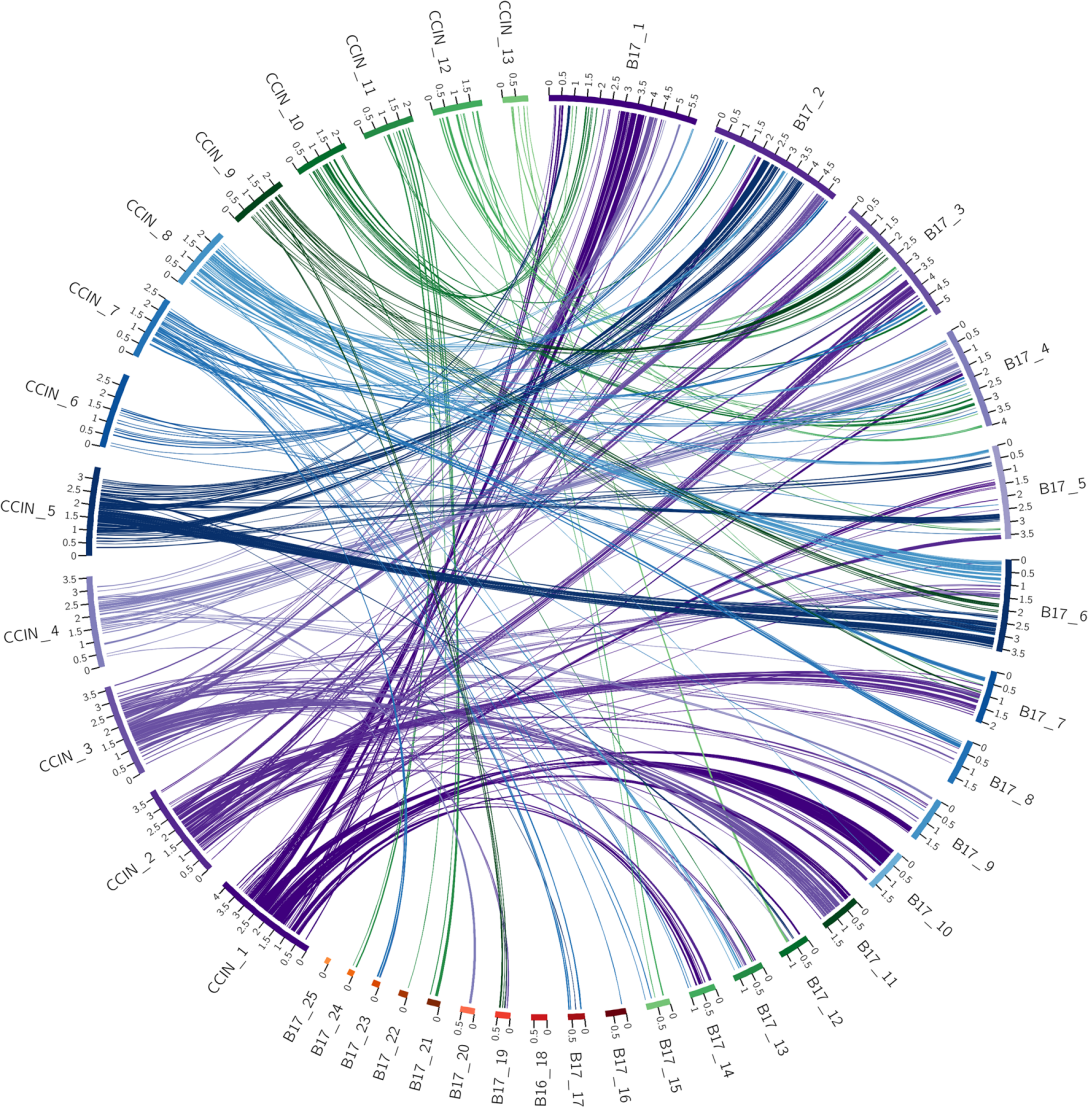
Circos plot of shared syntenic regions between *C. cinerea* and *L. edodes* B17. Syntenic regions are connected with colored ribbons scaled to the size of the syntenic regions. These ribbons match the colors of the *C. cinerea* scaffold blocks. Genomic scaffold lengths are in Mb and are indicated by the numerals on each scaffold.

The SynChro results in Fig. 1 show a high degree of conserved macrosyntenic structure. For example, scaffold 1 in the *C. cinerea* genome primarily links with scaffold 4 and 9 of the *L. novae-zelandiae* ICMP 18003 genome assembly. Of significance is that there are only four small ribbons linking scaffold 4 of the *L. novae-zelandiae* ICMP 18003 genome assembly to scaffold 7 of the *C. cinerea* genome assembly and no other links to scaffold 9. In contrast to this, Fig. 2 shows the macrosyntenic relationship between *C. cinerea* and *L. edodes* B17, with scaffold 1 of the *C. cinerea* genome assembly linking with eight scaffolds in the *L. edodes* B17 genome assembly, each of which has numerous links to other scaffolds within the *C. cinerea* genome assembly. Furthermore, the ordering of ribbon links within the *L. edodes* B17 genome assembly shows a high level of disorder, evidenced by 16 of the 25 scaffolds having ribbon links back to at least two or more *C. cinerea* assembly scaffolds. The pattern observed in Fig. 1 is representative of a high level of conserved macrosynteny, whereas the pattern observed in Fig. 2 shows a low level of conserved macrosynteny.

**Figure 3:**
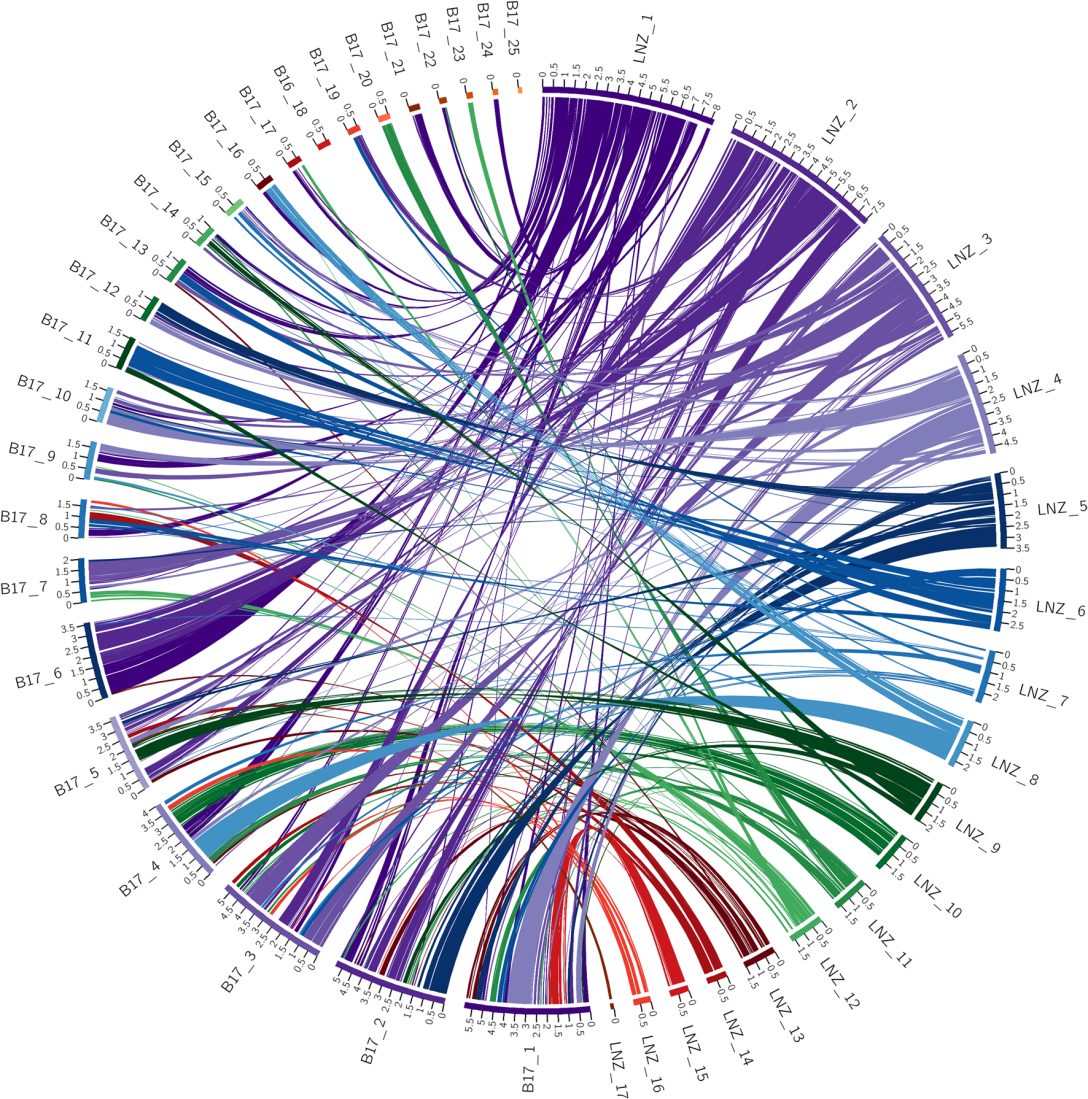
Circos plot of shared syntenic regions between *L. edodes* B17 and *L. novae-zelandiae* ICMP 18003. Syntenic regions are connected with colored ribbons scaled to the size of the syntenic regions. These ribbons match the colors of the *L. novae-zelandiae* ICMP 18003 scaffold blocks. Genomic scaffold lengths are in Mb and are indicated by the numerals on each scaffold.

Syntenic relationships as identified with SynChro between the *L. novae-zelandiae* ICMP 18003 genome assembly and the *L. edodes* B17 genome assembly are shown in Fig. 3. Despite SynChro finding the highest number of syntenic blocks between these species out of all pairwise comparisons, the macrosyntenic structure is massively disordered when compared with that seen in Fig. 1. Of the 25 scaffolds within the *L. edodes* B17 genome assembly, 17 have significant links to two or more scaffolds within the *L. novae-zelandiae* ICMP 18003 genome assembly. Some of these links may simply be a signal that they are part of the same chromosome but were unable to be assembled together due to the bioinformatics processes used and/or the nature of the dataset. However, scaffolds 1 to 6, all of which are 3.5 Mb or more, appear to have a high degree of macrosyntenic restructuring. Scaffold 1 for example, has links to 9 scaffolds within the *L. novae-zelandiae* ICMP 18003 genome assembly. Given that scaffold 2 of the *L. novae-zelandiae* ICMP 18003 assembly is a fully assembled telomere to telomere chromosome it represents a powerful data resource in this context. Interestingly, scaffold 6 of the *L. edodes* B17 genome has almost equal syntenic links to scaffold 1 and scaffold 2 of the *L. novae-zelandiae* ICMP 18003 genome assembly.

Analysis with Satsuma2 identified the same trend of macrosyntenic relationships as SynChro did, as evidenced by the Circos plots produced from the results. Overall Satsuma2 found significantly fewer macrosyntenic blocks than SynChro did; however, the pattern of organisation of those blocks is consistent with those found by SynChro. A composite image of all pair-wise syntenic analyses with both SynChro and Satsuma2 can be found in Supplementary Image 1.

## Discussion

In this study a chromosome-scale genome assembly for *L. novae-zealandiae* ICMP 18003 was created using ONT long-read data, illumina short-read data, and state-of-the-art bioinformatics tools. This assembly was produced using a bespoke hybrid assembly pipeline that has resulted in the first chromosome-scale genome assembly for the *Lentinula* genus. The *L. novae-zelandiae* ICMP 18003 genome assembly was used as a focal data set to conduct macrosyntenic analyses between it, *L. edodes*, and two other chromosome-scale genome assemblies from the taxonomic class Agaricomycota to identify whether there were any signals of domestication within the *L. edodes* lineage.

When considering the known history and biogeography of *L. edodes* and *L. novae-zelandiae,* the obvious feature that marks a difference between the two closely related species is the long history of cultivation across a large geographic region of *L. edodes.* This cultivation has spanned an estimated 1000 years, whereas *L. novae-zelandiae* has no history of cultivation and is a geographically isolated monophyletic lineage^1,39,40^. This history and how closely related these species are makes *L. novae-zelandiae* an ideal species against which to compare *L. edodes*.

Research on fungal genome structure in response to domestication suggests large macrosyntenic rearrangements can occur frequently and rapidly within populations and can even lead to diversification of lineages^7–9,11,13,31,41^. For example, research in brewer’s yeast has shown that chromosomes can fragment and subsequently be misrepaired by being fused together at telomere regions, resulting in new chromosomes with embedded telomeric sequences within them^11,13,41^. The results presented here identified telomeric regions embedded within scaffolds of the *L. edodes* genomes with no assembly gaps on either side. Given the high-quality nature of the genome and the lack of assembly gaps, the location of these telomeric regions is highly supported. Further potential signal of domestication is readily apparent in the high degree of macrosyntenic rearrangements that has occurred within the *L. edodes* B17 genome. Macrosyntenic analysis results from both SynChro and Satsuma2 show the same high level of conserved macrosyntenic structure shared between all genome assemblies assessed except for the *L. edodes* B17 genome. Previous research has found a similar level of conserved macrosynteny across species within the Agaricales^30,36^. The trend of conserved macrosynteny is supported by these results for all species except for *L. edodes*, with even the distantly related *P. noxium* from the basal order Hymenochaetales conforming to the trend. The B17 *L. edodes* genome assembly used within this study was derived from a single spore monokaryotic strain and as such some of its genomic structure may be due to inherited CLP’s from the parental strains^15–17^. The research in this area suggests that these CLP’s are derived from recombination of homologous chromosomes of differing lengths. If the parental strains of the B17 monokaryon had CLP’s then this may explain some of the structural differences observed, but it cannot explain the embedded telomeric regions nor the high degree of observed restructuring between scaffolds 1 through 6 identified via SynChro and Satsuma2.

The finding of such a large degree of macrosyntenic differences between these two species within the same genus is therefore remarkable. Furthermore, these results could explain the varying results produced in the linkage group analysis work previously done, where between 11 and 14 linkage groups have been found. It may be that these results are not an artifact of the protocols used but are simply due to different cultures having different karyotypes.

The biological ramifications of these genomic restructuring events are unknown, yet it is possible that these macrosyntenic changes have been underpinning adaptation of *L. edodes* to the commercial production environment. Future work in this area should focus on assessing the macrosyntenic differences within a large number of *L. edodes* cultivars through whole-genome sequencing. Research has shown that contiguity of a genome assembly is critical for meaningful macrosyntenic analysis, as such future assemblies should ideally be produced de novo using third-generation long-read sequencing technology with a robust quality-controlled assembly pipeline that involves a final curation step^42^. This final curation step is highly recommended^43^ but rarely undertaken in contemporary genome assembly projects. Studies have identified many genome assemblies across all organismal groups that have significant amounts of contaminant sequence^44–49^. It would be prudent to investigate the gene regions flanking these structural re-arrangements as these may have given rise to fusion genes, pseudogenes or differentially regulated genes. Further validation of these structural rearrangements with wet-lab based techniques such as karyotyping and PCR amplification of fusion genes would provide strong evidence to support these findings.

With only a single highly-contiguous *L. edodes* genome available, it is beyond the scope of this research to report on how much macrosyntenic diversity exists within the population of *L. edodes*. It is reasonable to hypothesize that there may be significant macrosyntenic variation within the *L. edodes* population, and it is likely these differences would be readily found between wild populations and cultivars that are commercially used. The genome assembly of *L. novae-zelandiae* reported here will provide a valuable resource for researchers undertaking comparative genomic studies within the genus *Lentinula* as well as for those with an interest in exploring the effects of domestication on fungi.

## Conclusion

The highly contiguous genome assembly of *L. novae-zelandiae* produced here has provided the means to make a meaningful macrosyntenic comparison between it and *L. edodes*. This comparison has revealed a large degree of macrosyntenic re-structuring within the B17 genome assembly of *L. edodes* that is potentially due to domestication. The genome assembly of *L. novae-zelandiae* is the first chromosome-scale assembly for the *Lentinula* genus and as such it represents a rich resource for future research; not only this but the methodology presented here provides a means for the production of high-quality fungal reference genomes using state-of-the-art technology.

## Methods

### Fungal culture

A culture of *Lentinula novae-zelandiae* was obtained from the International Collection of Microorganisms from Plants held by Manaaki Whenua – Landcare Research in New Zealand^50^. This axenic culture was isolated from a fruiting body collected in the Dunedin Botanic Gardens in 1991 by Dr Peter Buchanan and was deposited as ICMP 18003.

### *Lentinula novae-zelandiae* ICMP 18003 DNA extraction and sequencing

Fungal genomic DNA was extracted from the dikaryotic culture ICMP 18003 of *Lentinula novae-zelandiae* that had been grown on PDA media at 23 °C for two weeks. Fungal tissue was ground into a fine powder using liquid nitrogen in a mortar and pestle before being extracted using the phenol/chloroform based protocol: High-quality DNA from fungi for long-read sequencing protocol^51^ DNA purity was assessed on a Nanodrop spectrophotometer. DNA fragmentation was assessed by running 1 ul of DNA extract on a 1% agarose gel at 80 V for 120 min. Concentration of DNA was assessed using the dsDNA HS assay on a Qubit 4 fluorometer (Thermo Fisher).

### Illumina library preparation and sequencing

A library was prepared from the extracted DNA by Macrogen using the TruSeq Nano DNA kit with a 350 bp insert size. This was then sequenced on an illumina MiSeq platform with 100 bp paired end reads.

### DNA Size selection and library preparation

DNA fragments less than 10 kb were depleted via a size selection protocol using AMPure XP beads (Beckman Coulter). 0.45X volume of resuspended AMPure XP bead solution was added to the extracted DNA and incubated at room temperature with gentle mixing for 20 min. The sample was then placed onto a magnetic rack until the solution was clear, following which the supernatant was removed and the sample was then washed twice with 200 ul of fresh 70% EtOH. DNA was then eluted in 50 ul of EB (10 mM Tris pH 8.0) at room temperature for 2 min before being returned to the magnetic rack until the solution was clear. The DNA containing supernatant was then transferred to a fresh 1.5-ml Eppendorf DNA LoBind tube.

DNA repair (NEBNext FFPE DNA Repair Mix, NEB M6630) was performed on extracted fungal genomic DNA following Oxford Nanopore Technologies recommended protocol. The repaired DNA was then purified by adding 60 ul of resuspended AMPure XP beads to the sample in a fresh 1.5 ml Eppendorf DNA LoBind tube. The sample was incubated at room temperature for 5 min with gentle mixing, washed twice with 200 ul fresh 70% ethanol, pellet allowed to dry for 30 s, and DNA eluted in 61 ul of EB (10 mM Tris pH 8.0). A 1 ul aliquot was quantified by fluorometry (Qubit 4) to ensure ≥ 1 ug DNA was retained.

Ligation was performed by adding 25 ul of Ligation Buffer, 10 ul of NEBNext Quick T4 DNA Ligase and 5 ul Adapter Mix (SQK-LSK109 Ligation Sequencing Kit, Oxford Nanopore Technologies (ONT)) to the 60 ul of DNA sample from the previous step. This was mixed gently and incubated at room temperature for 10 min.

The adaptor-ligated DNA was cleaned using 40 ul of AMPure XP beads and Short Fragment Buffer (SQK-LSK109). The purified-ligated DNA was resuspended in 15 ul EB (10 mM Tris pH 8.0), incubated at room temperature for 10 min, pelleting the beads again, and transferring the supernatant to a new tube. A 1-ul aliquot was quantified by fluorometry (Qubit 4) to ensure ≥ 500 ng DNA was retained.

### MinION sequencing

MinIon sequencing was performed as per manufacturer’s guidelines using a single R9.4 flow cell (FLO-MIN106D). MinION sequencing was controlled using Oxford Nanopore Technologies MinKNOW software.

### Genome assembly and annotation of* Lentinula novae-zelandiae* ICMP 18003

#### Genome size and heterozygosity estimation

Genome size, repetitive content and heterozygosity were estimated using the illumina sequence data set and the online web tool GenomeScope^52^. Kmer counting was done using Jellyfish v. 2.2.10^53^ with the resulting histogram uploaded to the GenomeScope server for analysis.

#### DNA sequence processing and quality control

The illumina short-read data set was trimmed using Sickle v. 1.33 in the paired-end mode^54^. Raw minion signal data was basecalled using Guppy v. 3.3.0 using the high accuracy dna_r9.4.1_450bps_hac.cfg model with homopolymer correction and a qscore filter set to a minimum of 6. The basecalled long-read MinION data was trimmed using Porechop v. 0.2.4 (Wick, R)^55^ with default parameters. The trimmed long-read dataset was corrected using NECAT v. 20200119^56^ with an estimated genome size of 60 M with all other settings as default.

#### Genome assembly and quality control

The NECAT corrected long-read MinION data set was assembled using Canu v. 2.0^57^. The assembled sequences were polished using HyPo v. 1.0.2^58^ with the trimmed illumina short-read data set and the NECAT corrected MinION data set. The genome assembly was then phased using the Purge Haplotigs v. 1.1.1 pipeline^59^. Scaffolding of the assembled sequences was undertaken with SLR v. 1.0^60^ using the NECAT corrected long-read MinION data set. SLR was run with default settings.

Curation of the genome was undertaken using Tapestry v. 1.0.0^61^, with a read depth subsampling of 50X coverage. Contaminant or residual duplicated sequences were removed when they had a read depth coverage that fell outside the range of 30–50X coverage, or with a GC content of less than 45%. These parameters were selected based on recommendations from the Tapestry documentation and the GC content of the *L. edodes* genomes. Following this curation, the assembly was polished a final time using Pilon v. 1.23^62^ using the illumina short-read data set. Core genome metrics were assessed using QUAST with default settings, v. 5.0.2^63^. Genome completeness was assessed using BUSCO v. 4.0.2^64^ with the agaricales_odb10.2019-11-20 dataset and *Coprinus* as the Augustus species in the genome mode.

#### Genome annotation

RepeatModeler v. 2.0.1^65^ was used to produce a custom repeat database for each *Lentinula* genome assembly with the -LTRStruct option. The resultant repeat database was used with RepeatMasker v. 4.1.0^66^ to mask repeats in each genome assembly.

The Funannotate v. 1.7.4^67^ pipeline was used to predict gene sets for the repeat masked *Lentinula* genome assemblies, with a minimum protein length of 20 amino acids. Transcript evidence to be used for downstream gene model prediction was created using Trinity v. 2.8.5^68^. The following datasets were downloaded from NCBI Genbank with SRAtoolkit 2.9.6: SRR527823, SRR5891391, SRR5891392, SRR5891393. Each dataset was aligned to the *L. edodes* B17 genome using STAR v. 2.7.0^69^. Each dataset was assembled using Trinity in the genome guided assembly mode with the jaccard clip option turned on and a max intron size set to 75^68^. Funannotate used the assembled RNAseq data and the Basidiomycota BUSCO ODB9 dataset to train the ab initio gene prediction programs Augustus v. 3.3.3^70^, GeneMark-ET v. 4.0^71^ and GlimmerHMM v. 3.0.4^72^. EVidence Modeler v. 1.1.1^73^ was used to generate a consensus set of gene predictions from the ab initio prediction programs. In the training step, the four assembled transcriptome datasets previously described were used as well as the UniProt/SwissProt protein database^74^. tRNAscan-SE v. 2.0.5^75^ was used to predict tRNA genes.

### Comparative analysis

#### Agaricomycete reference genomes

The NCBI GenBank genome database was searched to identify *L. edodes* genomes appropriate for this study as well as to identify chromosome-scale genome assemblies within the taxonomic class Agaricomycetes. This was done by searching for “Agaricomycetes”, with subsequent manual parsing of the summary file produced. Assemblies identified as chromosome scale were flagged for further analysis with QUAST and had their telomeric regions identified. Assemblies that had predicted gene sets were identified as suitable for downstream macrosynteny analysis.

#### Macrosynteny analysis

To analyse the macrosyntenic relationships between the different genome assemblies thoroughly and reliably, macrosynteny analysis was conducted using two different pieces of software, each of which takes a different approach to identifying macrosyntenic relationships.

The January 2015 version of the SynChro package from the CHROnicle software suite^76,77^ was used with a delta value of 2. This software aims to identify homologous gene regions and takes as input the assembled genomes and a set of gene predictions for each assembly. In parallel to this, genome synteny analysis was also conducted with Satsuma2 v.20161123^78^. This software aims to identify homologous nucleotide regions and takes as input the assembled genome. To visualize the output from these software, Circos v. 0.69-8^79^ was used.

## Supplementary Information

Supplementary Information.

## Data Availability

The genomic data generated and analysed during this study are available at the Joint Genome Institute: https://mycocosm.jgi.doe.gov/LnoICMP18003A_1/LnoICMP18003A_1.home.html.
