# Supplementary material for: Macrosynteny analysis between Lentinula edodes and Lentinula novae-zelandiae reveals signals of domestication in Lentinula edodes
Source: Sci Rep. 2021 May 10;11:9845. doi: 10.1038/s41598-021-89146-y (PMC8110776; doi:10.1038/s41598-021-89146-y)
Supplement: Supplementary file 1 — Supplementary Information. [file 41598_2021_89146_MOESM1_ESM.pdf]

Macrosynteny analysis between *Lentinula edodes* and *Lentinula novae-zelandiae* reveals signals of domestication in *Lentinula edodes*

Supplementary tables and figures

Corresponding author: Christopher Alan Smith, Manaaki Whenua – Landcare Research

	# contigs	Largest contig	Total length	GC (%)	N50	# N's per 100 kbp	Telomeric regions
A. bisporus	13	3,550,205	30,417,844	46.5	2,550,681	428.52	0
A. ostoyae	11	6,974,893	56,666,185	48.37	5,329,788	1200.01	19
C. cinerea	68	4,146,986	36,192,590	51.64	3,468,139	0.09	0
F. velutipes	11	5,535,272	35,642,541	49.79	3,897,418	1050.86	2
H. erinaceus	45	6,077,030	41,205,484	52.43	3,208,415	1.7	3
L. edodes B17	31	5,849,119	46,106,741	46.15	3,662,990	425.45	5
L. novae zelandiae ICMP 18003	17	8,122,969	48,944,544	46.49	4,832,147	56.57	15
P. ostreatus	89	4,773,661	34,364,324	50.91	2,851,238	797.62	4
P. noxium	13	4,533,208	31,607,963	41.57	2,736,193	11.1	13
T. hirsuta	13	4,437,623	37,433,966	56.66	3,045,029	80.14	26

Supplementary table 1: Genome assembly metrics

Genome 1 (G1)	*C. cinerea* CC3	*C. cinerea* CC3	*L. edodes* B17
Genome 2 (G2)	*L. edodes* B17	*L. novae-zelandiae*	*L. novae-zelandiae*
Number of chromosomes G1	13	13	29
Number of chromosomes G2	29	17	17
Number of genes in G1	13,231	13,231	11,721
Number of genes in G2	11,721	12,099	12,099
Average similarity between syntenic homologs %	63.80%	65.20%	86.20%
Number of synteny blocks	550	662	361
Number of times where 2 consecutive blocks along G1 are on the same chromosome in G2	285	517	166
Number of times where 2 consecutive blocks along G2 are on the same chromosome in G1	416	573	170
Average number of genes per block	6.7	6	28.9

Supplementary table 2: SynChro analysis results showing genome metrics and syntenic block metrics between pairwise comparisons of input genomes.

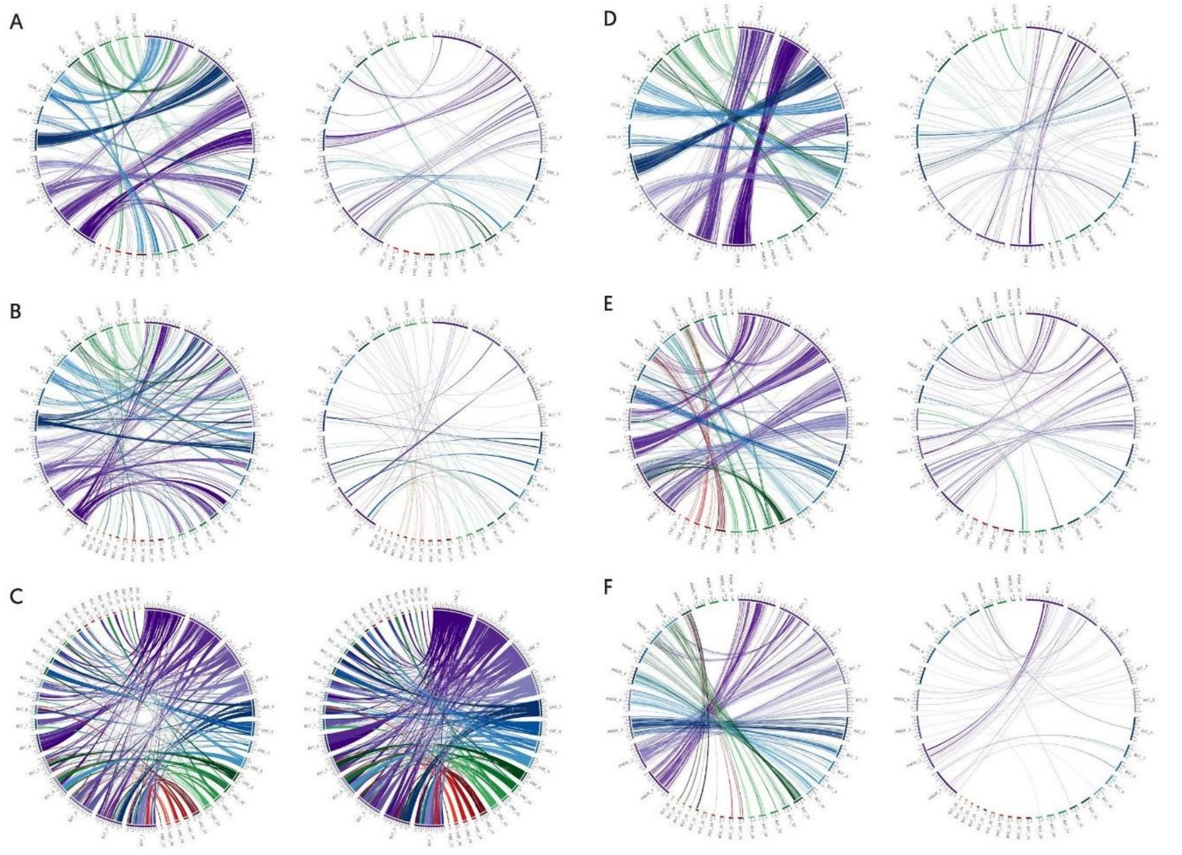

Supplementary Figure 1: Sets of Circos plots showing shared syntenic regions between pairs of genome assemblies as identified by SynChro (left) and Satsuma (right). Syntentic regions are connected with colored ribbons scaled to the size of the syntenic regions. (A) Synteny between C. cinerea and L. novae-zelandiae. (B) Synteny between C. cinerea and L. edodes. (C) Synteny between L. edodes and L. novae-zelandiae. (D) Synteny between C. cinerea and P. noxium. (E) Synteny between P. noxium and L. novae-zelandiae. (F) Synteny between P. noxium and L. edodes.
